# Assessment of TLR3-TRIF-NF-κB Innate Immune Signaling Pathway Induced by Newcastle Disease Virus and Construction of TLR3-Deficient BHK-21 Cells

**DOI:** 10.3390/v18101091

**Published:** 2026-10-02

**Authors:** Bingmei Dong, Jing Cai, Haiqi Zhao, Pengyan Gao, Qingqing Xu, Weiqin Meng, Na Tang

**Affiliations:** 1 College of Life Science, Cangzhou Normal University, Cangzhou 061001, China; dbmczsf2022@caztc.edu.cn (B.D.); czsygpy@163.com (P.G.); 2 Cangzhou Animal Disease Prevention and Control Center, Cangzhou 061001, China; txwy562520@163.com; 3 CasMed Biotech Co., Ltd., Baoding 071000, China; zhaohaiqi88888@163.com; 4 Shandong Binzhou Animal Husbandry and Veterinary Sciences, Binzhou 256600, China; zjzlxqq@126.com

**Keywords:** innate immune, TLR3, signaling pathway, Newcastle disease virus

## Abstract

The innate immune system plays a significant role in inhibiting viral infection and proliferation. To explore the signaling pathway of the innate immune response induced by Newcastle disease virus (NDV) in BHK-21 cells, the mRNA expression levels of pattern recognition receptors such as TLR3, TLR7, TLR8, and NOD2, as well as those of cytokines including TNF-α, IL-6, IL-1β, IL-10, IL-17, TRIF, TRAF3, and NF-κB were determined by transcriptome sequencing, qRT-PCR, and ELISA. All these results indicated that the NDV LaSota strain primarily induces the TLR3-TRIF-NF-κB signaling pathway in BHK-21 cells. Subsequently, an NF-κB inhibitor, a TLR3 inhibitor, and a TRAF3 inhibitor were separately used, and the viral titer was measured during the NDV LaSota strain infection of BHK-21 cells. The results displayed that the inhibition of TLR3 or NF-κB effectively increased the viral titer of NDV. Hence, the CRISPR/Cas9 system was used to create a BHK-TLR3^−^ cell clone in which TLR3 was knocked out, and the cells were assessed for morphology, cell density, cell viability, and genetic stability. Finally, the cells were infected with the NDV LaSota strain. The results showed that the BHK-TLR3^−^ cell line is a stable cell line with markedly enhanced NDV LaSota strain production. Furthermore, the knockout of the TLR3 gene significantly inhibited the expression of IFN-α, IFN-β, IL-1β, and TNF-α, especially that of IL-1β and TNF-α. In summary, the present research revealed the main signaling pathway of the innate immune response induced by the NDV LaSota strain and provides a promising basis for NDV production.

## 1. Introduction

Newcastle disease (ND) is an acute, febrile, and highly contagious infectious disease caused by Newcastle disease virus (NDV). It is widely spread worldwide and seriously endangers poultry farming [1,2,3]. Currently, the prophylactic vaccination of chicken flocks remains the primary strategy for controlling ND [4,5]. Up to now, the production of traditional ND vaccines has mainly relied on the inoculation of chicken embryos and harvesting allantoic fluid for preparation. This process faces many challenges, such as poor batch-to-batch stability, the risk of exogenous virus contamination, and the harmless disposal of chicken embryo waste. Among the potential alternatives to traditional egg-based poultry vaccine production, the use of cell lines has been extensively explored. At present, MDCK cells have been widely employed for influenza vaccine production [6]. In addition, cells such as BHK-21 cells, MDCK cells, and Vero cells have achieved full suspension culture for vaccine production [7,8]. Although NDV can grow in various cells, such as BHK-21 cells, Vero cells, and DF-1 cells, the generally low viral titer fails to meet the production requirements. This may be due to factors such as viral susceptibility and the innate immune response inhibiting the proliferation of NDV. Therefore, it is particularly urgent to effectively increase the viral titer of NDV in cells.

Innate immunity serves as the first line of defense for the host against viral invasion [9,10]. When pathogenic microorganisms infect host cells, the pathogen-associated molecular patterns can be rapidly recognized by a series of pattern recognition receptors (PRRs) of host cells. Subsequently, the activation of signaling pathways can stimulate the host cells to secrete some cytokines to resist viral infection [11,12,13]. Among them, the signaling pathways of the host cells against RNA viruses mainly include Toll-like receptors (TLRs)-dependent pathways and RIG-I-like receptors-triggered cytoplasmic pathways [14,15]. Specifically, the TLR3 and MDA5 receptors mainly recognize double-stranded RNA from viruses, including the double-stranded RNA produced by single-stranded RNA viruses during replication. The TLR7 and TLR8 receptors mainly recognize single-stranded RNA viruses [16,17]. The RIG-I receptor primarily recognizes viruses containing 5′ppp-RNA [18]. NDV is a single-stranded and negative-sense RNA virus, but it can generate double-stranded RNA during replication [19,20]. Therefore, all the TLR3, TLR7, TLR8, and MDA5 receptors can recognize NDV and transmit the relevant signals to inhibit viral replication [17,21]. It is reported that the expression levels of TLR3, TLR7, and MDA5 receptors significantly increase after NDV infects the DF-1 cells [22,23].

In a previous study, we demonstrated that BHK-21 cells are more suitable for NDV proliferation than HeLa, Vero, DF-1, and LMH cells [24]. Therefore, the BHK-21 cells were selected as the subject for the subsequent research. In order to increase the viral titer of NDV in the BHK-21 cells, we first determined the primary signaling pathway of the innate immune response induced by NDV. Moreover, it was demonstrated that inhibition of the signaling pathway can significantly promote the replication of NDV in BHK-21 cells. Therefore, we successfully knocked out the TLR3 gene of BHK-21 cells using the CRISPR/Cas9 system, thereby significantly increasing the viral yield in BHK-21 cells.

## 2. Materials and Methods

### 2.1. Cell Line and Virus

BHK-21 cells were purchased from the American Type Culture Collection (ATCC, Rockefeller, Manassas, VA, USA) and maintained in Dulbecco’s Modified Eagle Medium (DMEM, Sigma-Aldrich Corporation, St. Louis, MO, USA) supplemented with 6% fetal bovine serum (Sigma-Aldrich Corporation, St. Louis, USA) at 37 °C. The lentogenic NDV LaSota strain was obtained from the China Institute of Veterinary Drug Control (Beijing, China) and stored at −80 °C.

### 2.2. NDV Infection and Transcriptome Sequencing

BHK-21 cells were seeded into 6-well plates (1 × 10^6^ cells/well). When the cells grew to single layers, the culture medium was discarded and the NDV LaSota strain was inoculated at a multiplicity of infection of 0.5. After the cells were incubated for 1 h at 37 °C, the inoculum was removed and the DMEM containing 2 μg/mL TPCK-pancreatin (Sigma-Aldrich Corporation, St. Louis, USA) was added (2 mL/well). Then, the infected cultures were incubated at 37 °C with 5% CO_2_. At different times, the medium was discarded and cell lysis buffer was added (0.5 mL/well). Subsequently, the cells were harvested and subjected to transcriptome sequencing by Personalbio Biotech (Nanjing, China). The mRNA expression levels of PRRs and cytokines related to innate immunity were analyzed via expression difference analysis and KEGG enrichment analysis. Among them, at least two-fold changes were chosen as multiples of the difference to capture minor but functionally significant changes, and the differences were considered statistically significant at *p* < 0.05.

### 2.3. qRT-PCR Analysis

In order to verify the results of transcriptome sequencing, the total RNA was extracted with an RNA isolation kit (Accurate Biology, Changsha, China) after BHK-21 cells were infected with the NDV LaSota strain, and the RNA was reverse transcribed into cDNA by a reverse transcription kit (Takara, Beijing, China). Subsequently, the transcript levels of TLR3, TLR7, TLR8, NOD2, TRIF, NF-κB, and TRAF3 were analyzed using the Maxima SYBR Green qPCR kit (Accurate Biology, Changsha, China), and β-actin served as an invariant internal control. All the primers (Table 1) used in this study for qRT-PCR were selected from previous studies and synthesized by Sangon Biotech (Shanghai, China). In brief, the reaction mixture with a total volume of 10 μL contained 5 μL of SYBR Green mix, 5 μM of forward primer and reverse primer, 2 μL of cDNA, and 2 μL of RNase-free ddH_2_O. The following PCR conditions were utilized: 95 °C for 5 min, followed by 44 cycles of 95 °C for 10 s and 60 °C for 30 s. Subsequent melting curve analysis was performed to check the specific product.

### 2.4. ELISA Analysis

To verify the expression levels of the inflammatory factors, the BHK-21 cells infected with the NDV LaSota strain were harvested and centrifuged at 10,000 *g* for 5 min after freeze–thaw for three times. The supernatant was obtained, and the expression levels of IL-17, IL-6, IL-1β, IL-10, and TNF-α were determined by ELISA kits (Bioswamp^®^, Wuhan, China) according to the manufacturer’s instructions.

### 2.5. Employment of TLR3 Inhibitor, TRAF3 Inhibitor, and NF-κB Inhibitor

To explore whether the inhibition of the TLR3-signaling pathway can promote the proliferation of NDV in BHK-21 cells, DMEM containing an NF-κB inhibitor (7.5 μm/L), a TLR3 inhibitor (10 μm/L), or a TRAF3 inhibitor (7.5 μg/mL) was respectively put into 6-well plates after BHK-21 cells were infected with the NDV LaSota strain. When BHK-21 cells reached 90% confluency, the cells were harvested. Subsequently, the HA titer and the 50% tissue culture infectious dose (TCID_50_) were determined according to the Reed-Muench method.

### 2.6. Generation of BHK-21 Cells with TLR3 Knocked-Out via CRISPR/Cas9

Firstly, the single-guide RNA (sgRNA) targeting the TLR3 sequence was designed using a web-based sgRNA designing tool (http://crispor.gi.ucsc.edu/) (24 October 2024). Subsequently, the sgRNA was synthesized by General Biotech (Chuzhou, China) (Table 2). When BHK-21 cells were grown to 70–90% confluence in 24-well plates, the sgRNA was transfected into the BHK-21 cells with a one-step sgRNA transcription kit (Inovogen Tech. Co. Beijing, China). In brief, the CRISPR ribonucleoprotein complex was first prepared by mixing 2.5 μL of transcribed sgRNA with 2.5 μL of Cas9 protein, incubating for 10 min at room temperature. Next, 20 μL of CRISPR buffer was added to form the Cas9/sgRNA complex. Separately, the transfection reagent was prepared by combining 0.4 μL of Viromer with 24.6 μL of CRISPR buffer, and this mixture was rapidly combined with the Cas9/sgRNA complex in equal volumes. After incubation for 15 min at room temperature, the transfection mixture was added to the BHK-21 cells. 48 h post-transfection, the medium was discarded and the new complete medium containing puromycin (2 μg/mL) was put into the 24-well plates. The transfected cells were further cultured for 14 d at 37 °C and 5% CO_2_. Subsequently, the single-cell clones were isolated via the limiting dilution method in 96-well plates.

### 2.7. Identification of BHK-TLR3^−^ Cell Clone by RT-PCR and Sequencing Analysis

The total RNA of BHK-TLR3^−^ cell clone and BHK-21 cells were extracted and analyzed by RT-PCR with primers listed in Table 2. In brief, the reaction mixture with a total volume of 20 μL contained 10 μL of 2 × buffer, 15 μM of forward primer and reverse primer, 4 μL of cDNA, 4 μL of Taq DNA polymerase, and 2 μL of ddH_2_O. The following PCR conditions were utilized: 95 °C for 2 min, followed by 36 cycles of 95 °C for 30 s, 58 °C for 30 s, and 72 °C for 30 s, extended at 72 °C for 10 min. The full-length TLR3 gene from each cell clone was subjected to paired-end sequencing by General Biotech (Chuzhou, China). Subsequently, the resulting nucleotide and deduced amino acid sequences of the TLR3 mutants were compared with the wild-type TLR3 sequence to confirm the gene editing.

### 2.8. Western Blot Analysis

In order to determine the expression level of TLR3 protein in BHK-TLR3^−^ cells, the total protein of BHK-TLR3^−^ cells was extracted and separated by electrophoresis with 12% SDS-polyacrylamide gel. The resolved proteins were transferred to a 0.45 μm polyvinylidene fluoride membrane (Beyotime, Shanghai, China). The membrane was blocked with blocking buffer (Beyotime, Shanghai, China) at 37 °C for 1 h. After three washes, the membrane was incubated overnight with rabbit polyclonal anti-mouse TLR3 antibody at 4 °C (Abcam, Cambridge, England). Then, the membrane was washed and treated with horseradish peroxidase-conjugated goat polyclonal anti-rabbit IgG antibody (Beyotime, Shanghai, China) at 37 °C for 1 h. Next, the membrane was treated with luminescent solutions A and B. Finally, the sites of antibody–antigen reaction on the membrane were immediately detected using a chemiluminescence system. The target bands were densitometrically scanned to obtain the semiquantitative results. Relative levels of TLR3 protein were normalized to the β-actin in each corresponding data point.

### 2.9. Observation of BHK-TLR3^−^ Cell Morphology, Growth Kinetics, and Cell Viability

BHK-TLR3^−^ cells and BHK-21 cells were continuously passaged for five generations. At 24 h, 48 h, 72 h, and 96 h of each passage, the number of cells and the proportion of viable cells were determined using trypan blue exclusion. At the same time, the morphology of BHK-TLR3^−^ cells and wild-type BHK-21 cells was examined.

### 2.10. Evaluation of Genetic Stability of the TLR3 Mutant Gene in BHK-TLR3^−^ Cells

To investigate whether the TLR3 mutant gene undergoes any genetic reversion during continuous culture, the BHK-TLR3^−^ cells were continuously passaged for 20 generations. The TLR3 gene of BHK-TLR3^−^ cells was respectively amplified at the 0th, 5th, 10th, and 20th generations, and the similarity of the gene sequence was analyzed to assess the potential genetic reversion.

### 2.11. Proliferation of NDV LaSota Strain in BHK-TLR3^−^ Cells

To determine whether NDV can exhibit higher replication efficiency in BHK-TLR3^−^ cells, the BHK-TLR3^−^ cells and BHK-21 cells were separately inoculated with the NDV LaSota strain at a multiplicity of infection of 0.5. When the infected BHK-TLR3^−^ cells and BHK-21 cells grew to 90% confluency, the cell cultures were harvested. Subsequently, the viral titers and the expression levels of IFN-α, INF-β, IL-1β, IL-6, and TNF-α were determined.

### 2.12. Statistical Analysis

Data were analyzed using IBM SPSS Statistics 20.0, and statistical significance was assessed by one-way ANOVA. Data are presented as mean ± standard deviation, and differences were considered statistically significant at *p* < 0.05 or *p* < 0.01. All experiments were independently performed at least three times, with a minimum of three samples per group in each experiment.

## 3. Results

### 3.1. Focus on the Signaling Pathways of Innate Immunity After BHK-21 Cells Were Infected with the NDV LaSota Strain

**Figure 1:**
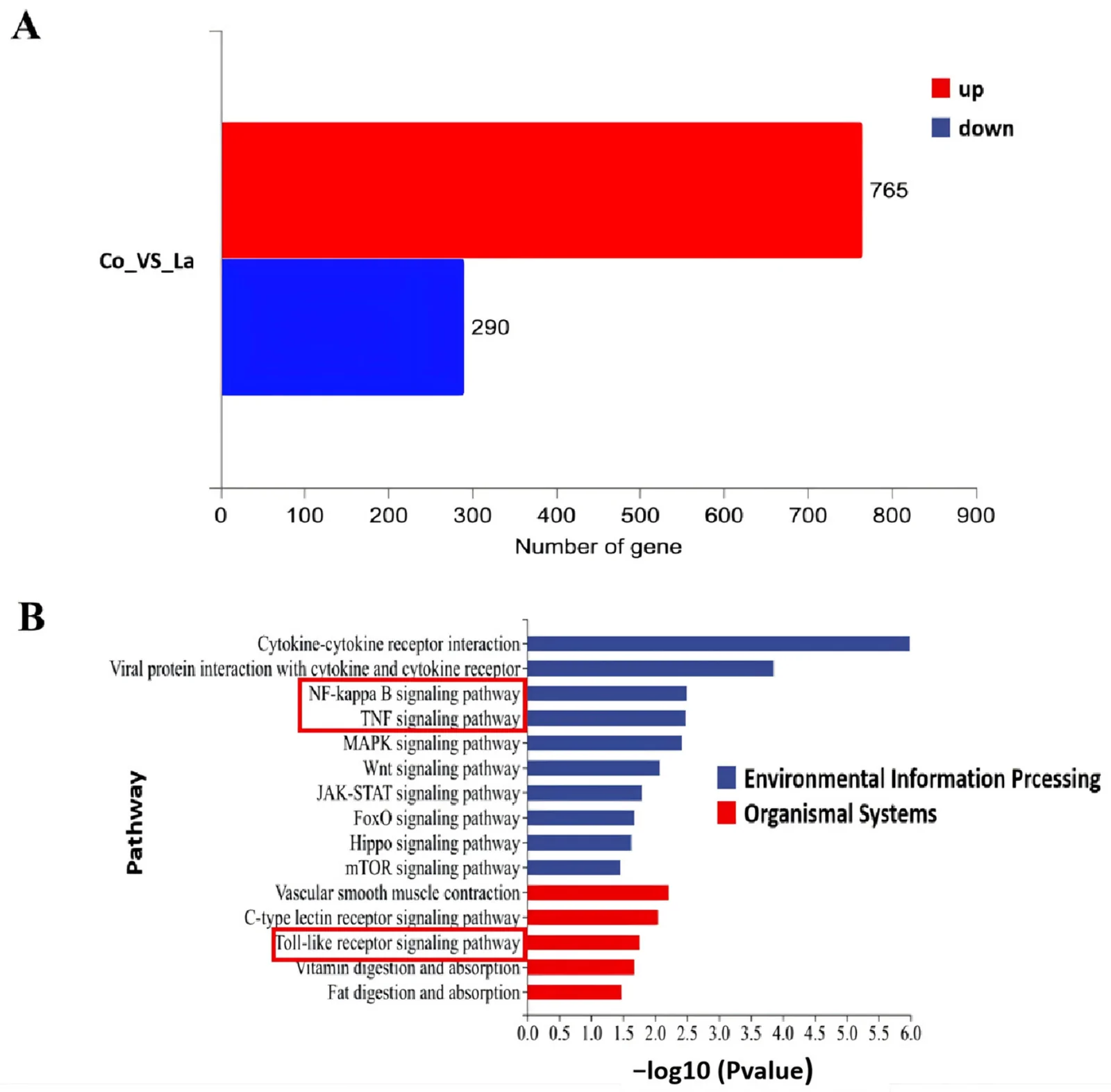
Transcriptome sequencing analysis after BHK-21 cells were infected by NDV LaSota strain. (**A**) The expression differences analysis of mRNA between BHK-21 cell control group (co) and the infection group of NDV LaSota strain (La). (**B**) The KEGG enrichment analysis of the up-regulated genes regarding signaling pathways. Note: The “Co_VS_La” in Figure 1A means that the BHK-21 cell control group is compared with the infection group.

To determine the main signaling pathway of innate immunity, transcriptome sequencing was carried out after BHK-21 cells were infected with the NDV LaSota strain. The differential expression analysis showed that the number of up-regulated genes and down-regulated genes in the NDV infection groups were 765 and 290, respectively (Figure 1A). Among all up-regulated genes, the KEGG enrichment analysis displayed that the signaling pathways related to innate immunity were mainly concentrated in the TLRs signaling pathway, the NF-κB signaling pathway, and the TNF signaling pathway (Figure 1B).

### 3.2. The mRNA Expression of TLR3, TLR7, TLR8, and NOD2 According to Transcriptome Sequencing

**Figure 2:**
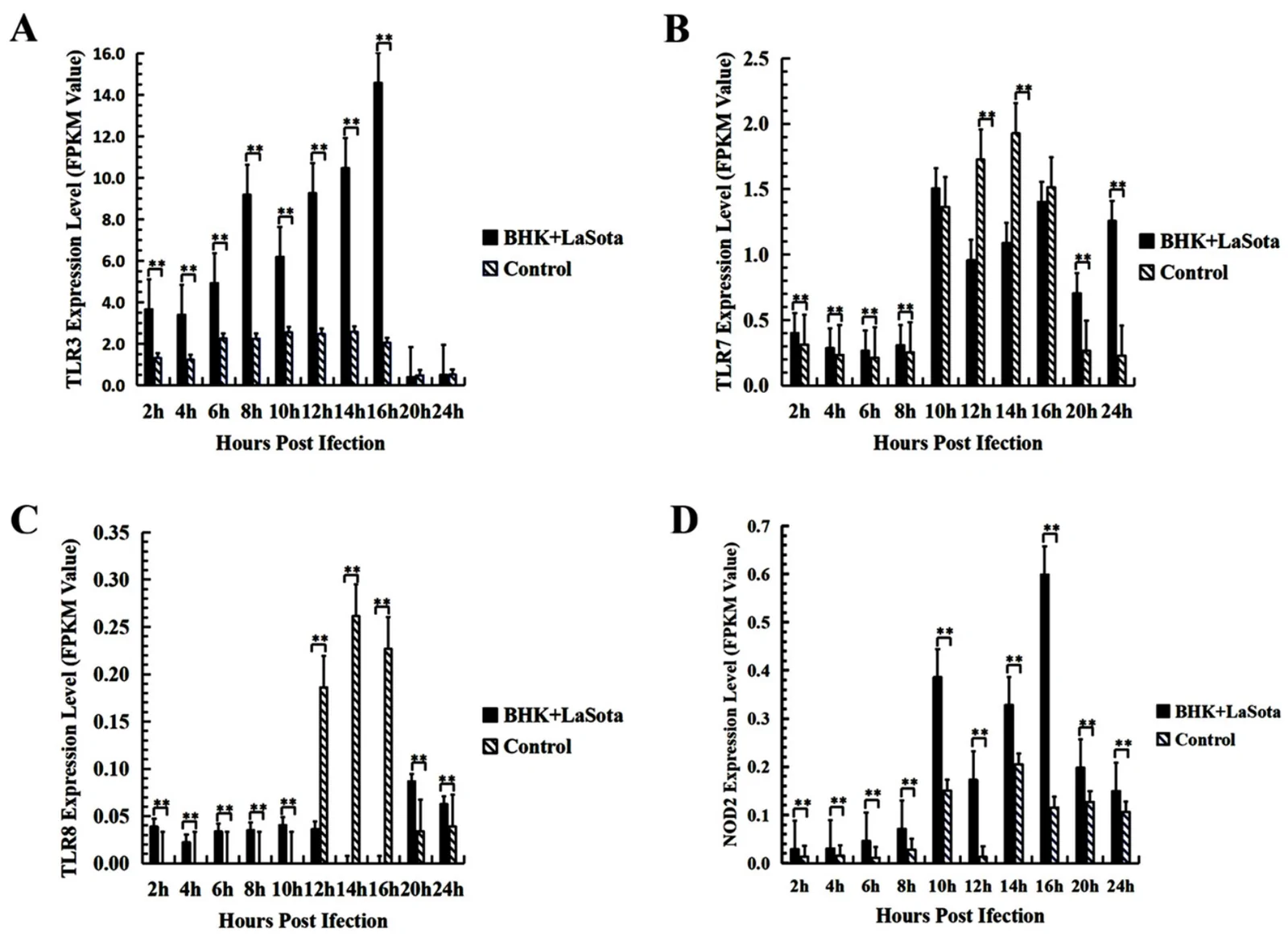
The mRNA expression of TLR3 (**A**), TLR7 (**B**), TLR8 (**C**), and NOD2 (**D**) according to transcriptome sequencing after BHK-21 cells were infected with NDV LaSota strain ** *p* < 0.01.

Regarding TLR3, from 2 h to 16 h post-infection, the mRNA expression levels of TLR3 in the infection groups were significantly higher than those in the control groups (*p* < 0.01) (Figure 2A). Regarding TLR7 and TLR8, although the mRNA expression levels of TLR7 and TLR8 in the infection groups showed significant differences from those in the control groups, they were sometimes significantly higher and sometimes significantly lower than those in the control groups, which indicated unstable expression (Figure 2B,C). Regarding NOD2, its mRNA expression levels in the infection groups were significantly higher than those in the control groups from 2 h to 24 h after BHK-21 cells were infected with the NDV LaSota strain (*p* < 0.01), but the overall expression levels were obviously lower than those of TLR3 (*p* < 0.01) (Figure 2D).

### 3.3. The mRNA Expression of Cytokines According to Transcriptome Sequencing

#### 3.3.1. TNF Signaling Pathway

**Figure 3:**
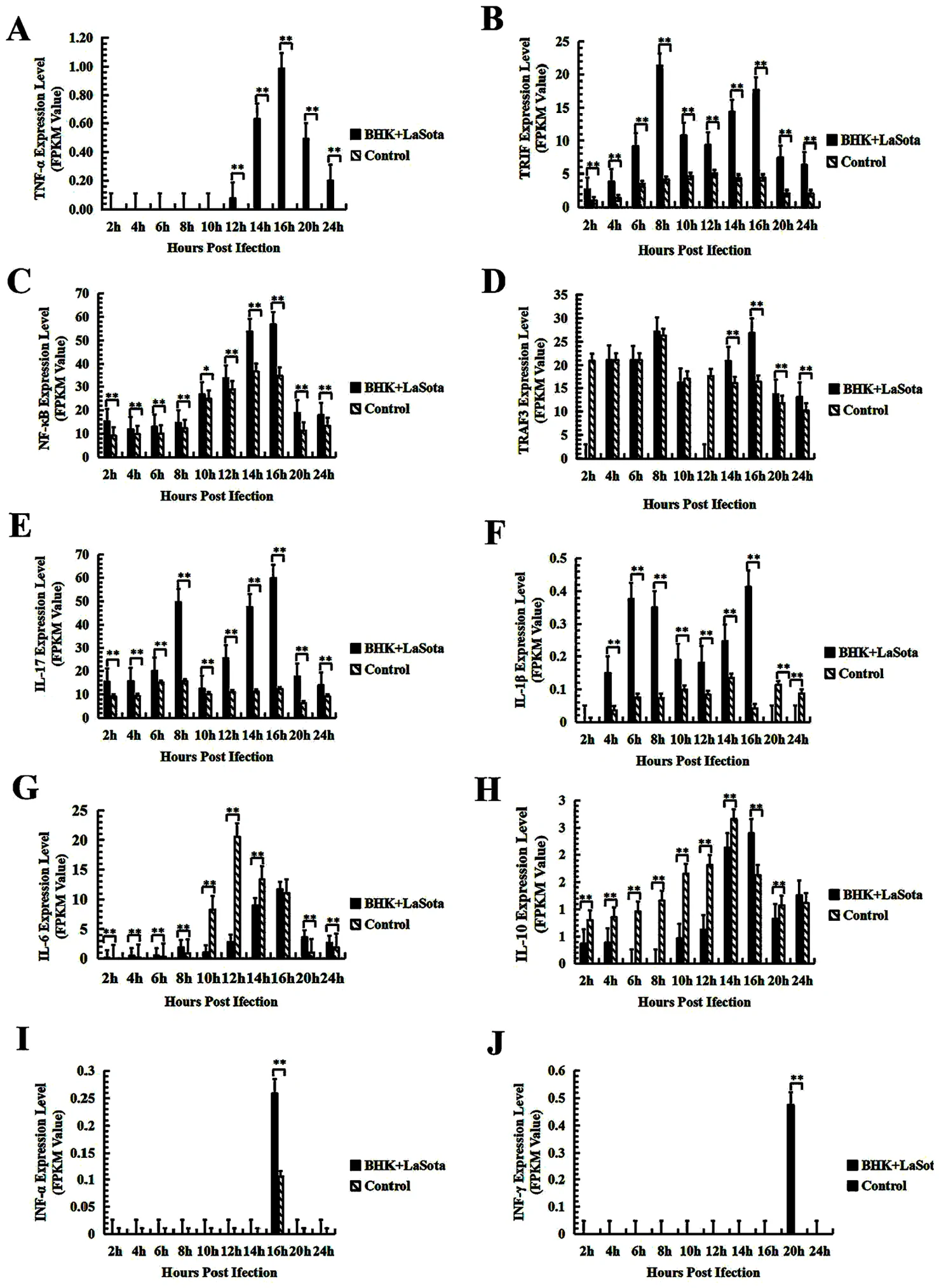
The mRNA expression of TNF-α (**A**), TRIF (**B**), NF-κB (**C**), TRAF3 (**D**), IL-17 (**E**), IL-1β (**F**), IL-6 (**G**), IL-10 (**H**), IFN-α (**I**), and IFN-γ (**J**) according to transcriptome sequencing after NDV LaSota strain infection of BHK-21 cells. * *p* < 0.05, ** *p* < 0.01.

Regarding the TNF signaling pathway, no changes were found in the mRNA expression levels of TNF-β. On the other hand, the TNF-α gene began to express at 12 h after BHK-21 cells were infected with the NDV LaSota strain, and its mRNA expression levels were significantly higher than those in the control groups from 12 h to 24 h (*p* < 0.01) (Figure 3A).

#### 3.3.2. Signaling Pathway Regulated by TLR3 Receptor

From 2 h to 24 h after BHK-21 cells were infected with the NDV LaSota strain, the mRNA expression levels of TRIF and NF-κB were significantly higher than those in the control groups (*p* < 0.01) (Figure 3B,C). However, the mRNA expression levels of TRAF3 displayed no significant differences between the infection groups and the control groups from 2 h to 12 h (*p* > 0.05). From 14 h to 24 h, the mRNA expression levels of TRAF3 in the infection groups were distinctly increased compared to those in the control groups (*p* < 0.01) (Figure 3D).

#### 3.3.3. Pro-Inflammatory Cytokines IL-17, IL-1β, and IL-6

Regarding IL-17, its mRNA expression levels in the infection groups were obviously higher than those in the control groups from 2 h to 24 h after BHK-21 cells were infected with the NDV LaSota strain (*p* < 0.01) (Figure 3E). On the other hand, the mRNA expression levels of IL-1β in the infection groups were higher than those in the control groups from 4 h to 16 h (*p* < 0.01), but they remarkably decreased from 20 h after infection compared with the control groups (*p* < 0.01) (Figure 3F). Regarding IL-6, its mRNA expression levels in the infection groups were obviously higher than those in the control groups from 2 h to 8 h and 20 h to 24 h after BHK-21 cells were infected with the NDV LaSota strain (*p* < 0.01), but they remarkably decreased from 10 h to 14 h compared with the control groups (*p* < 0.01) (Figure 3G).

#### 3.3.4. Anti-Inflammatory Cytokine IL-10

The results showed that the mRNA expression level of IL-10 in the infection group was obviously higher than that in the control group at 16 h (*p* < 0.01). However, from 2 h to 14 h and 18 h to 20 h, its expression level remarkably decreased in the infection group compared with the control group (*p* < 0.01) (Figure 3H).

#### 3.3.5. Antiviral Cytokines IFN-α, IFN-β, and IFN-γ

No changes were found in the mRNA expression levels of IFN-β. It was only at 16 h after infection that the mRNA expression level of IFN-α in the infection group was significantly higher compared with the control group (*p* < 0.01) (Figure 3I). Regarding IFN-γ, its mRNA expression level was obviously higher only at 20 h after BHK-21 cells were infected with the NDV LaSota strain compared with the control group (*p* < 0.01) (Figure 3J).

In sum, these results indicate that the primary signaling pathways of innate immunity are the TLR3-TRIF-NF-κB pathway and inflammatory response after BHK-21 cells are infected with the NDV LaSota strain. However, the antiviral immune response is inordinately suppressed in BHK-21 cells.

### 3.4. Check Analysis of the Expression Levels of TLR3, TLR7, TLR8, NOD2, TRIF, NF-κB, and TRAF3 by qRT-PCR

**Figure 4:**
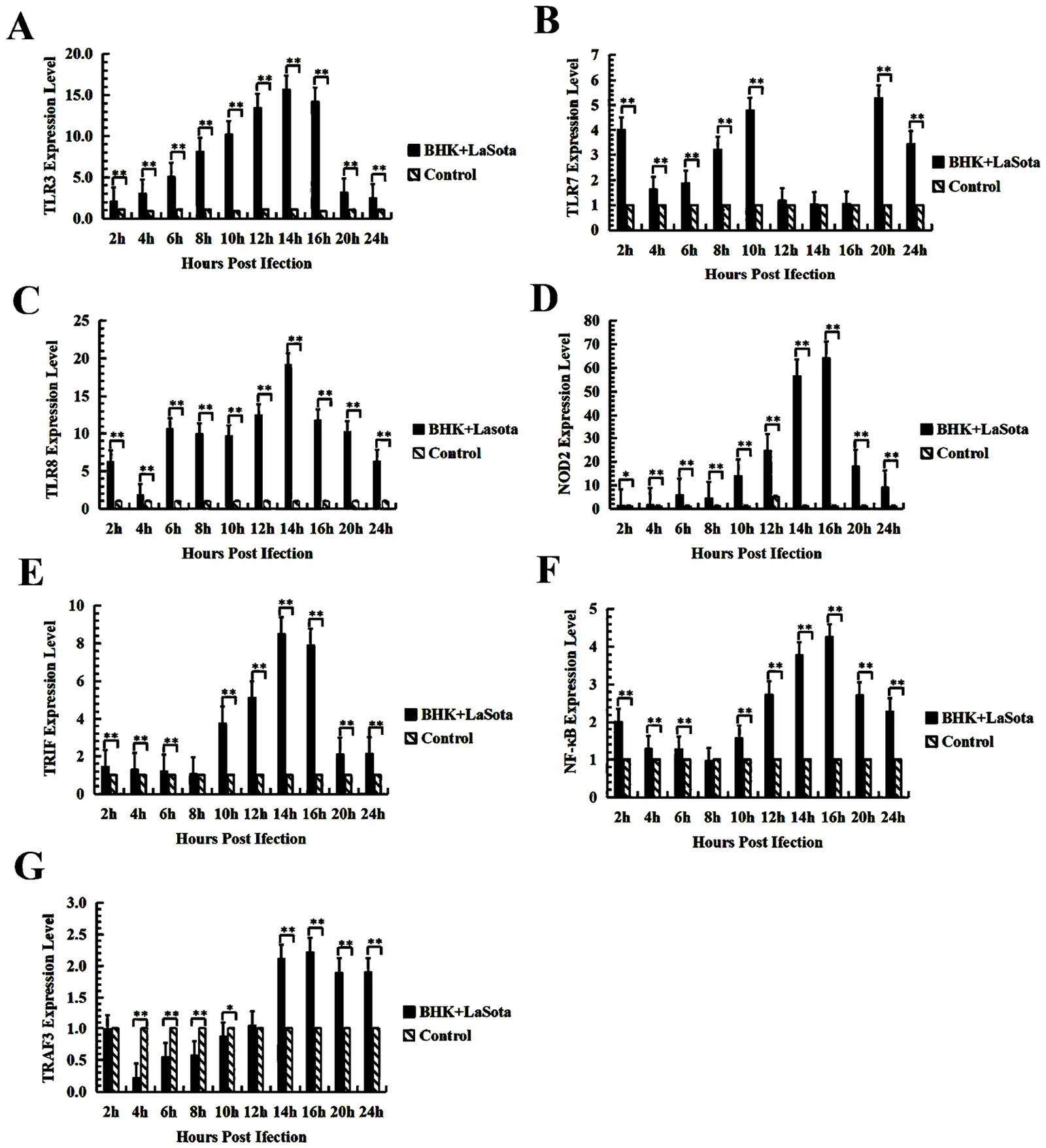
The expression levels of TLR3 (**A**), TLR7 (**B**), TLR8 (**C**), NOD2 (**D**), TRIF (**E**), NF-κB (**F**), and TRAF3 (**G**) were determined by qRT-PCR after BHK-21 cells were infected with the NDV LaSota strain. * *p* < 0.05, ** *p* < 0.01.

About TLR3, from 2 h to 24 h after BHK-21 cells were infected with the NDV LaSota strain, its expression levels significantly increased by 2.0 to 15.7 times (*p* < 0.01) (Figure 4A). About TLR7, similar to the results of transcriptome sequencing, from 2 h to 10 h and 20 h to 24 h after BHK-21 cells were infected with the NDV LaSota strain, the expression levels of TLR7 obviously increased by 1.6 to 4.8 times and 3.4 to 5.3 times (*p* < 0.01) (Figure 4B). About TLR8, unlike the results of transcriptome sequencing, its expression levels significantly increased by 1.8 to 19.2 times from 2 h to 24 h after BHK-21 cells were infected by NDV, but they never decreased (Figure 4C). About NOD2, from 2 h to 24 h after BHK-21 cells were infected with the NDV LaSota strain, its expression levels were significantly higher than those in the control groups (*p* < 0.05) (Figure 4D). About TRIF, compared with the control groups—except at 8 h—its expression levels obviously increased by 1.2 to 8.5 times in the infection groups (*p* < 0.01) (Figure 4E). About NF-κB, similar to TRIF, its expression levels obviously increased from 2 h to 24 h after BHK-21 cells were infected with the NDV LaSota strain (*p* < 0.01), except at 8 h (Figure 4F). About TRAF3, similar to the results of transcriptome sequencing, its expression levels significantly increased by 1.9 to 2.2 times from 14 h to 24 h after BHK-21 cells were infected with the NDV LaSota strain (*p* < 0.01). But unlike the results of transcriptome sequencing, the expression levels of TRAF3 in the infection groups significantly decreased compared with the control groups from 2 h to 10 h (*p* < 0.01) (Figure 4G).

### 3.5. ELISA Detection of IL-17, IL-6, IL-1β, IL-10, and TNF-α

**Figure 5:**
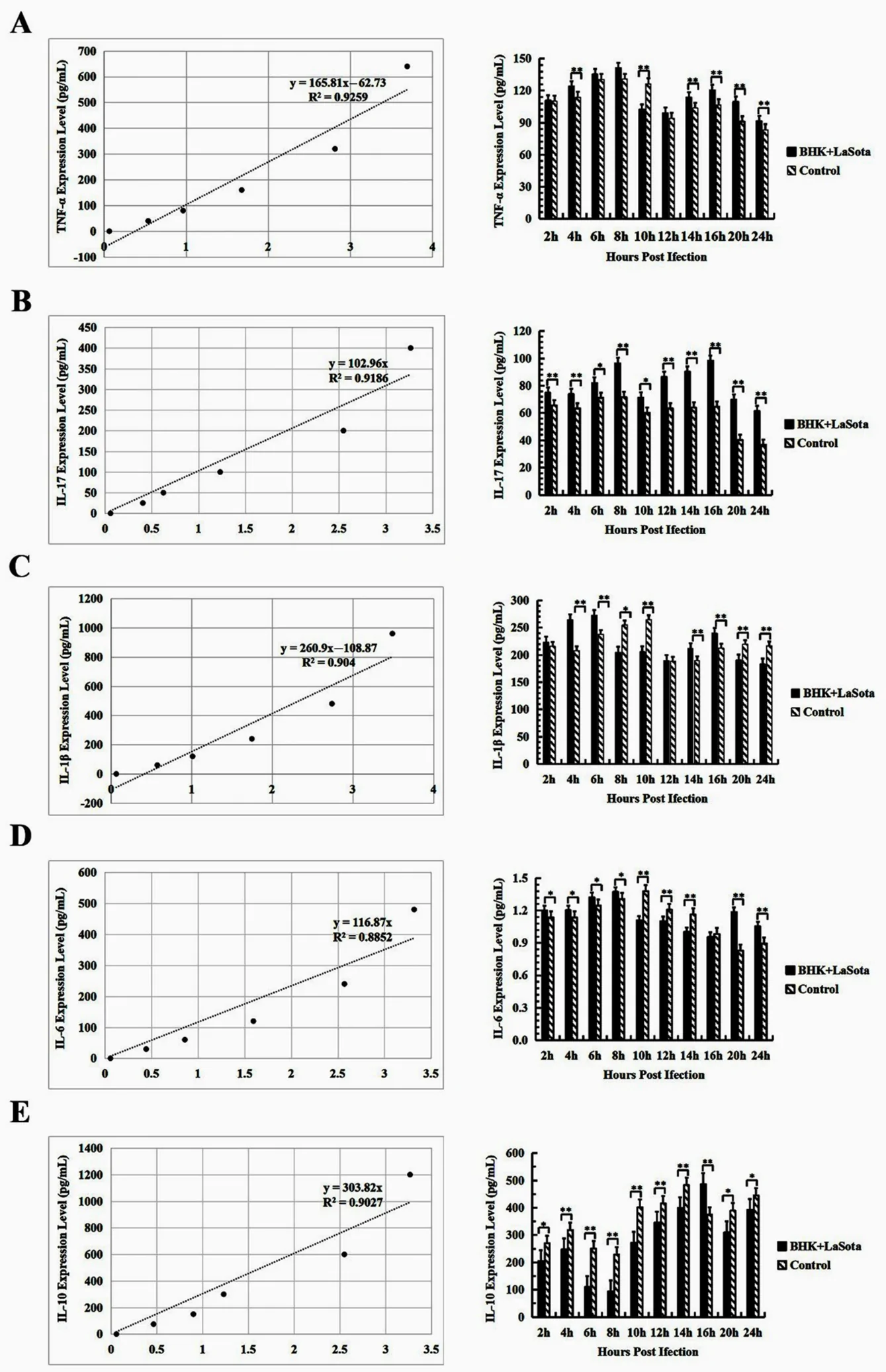
The ELISA analysis of TNF-α (**A**), IL-17 (**B**), IL-1β (**C**), IL-6 (**D**), and IL-10 (**E**) after BHK-21 cells were infected with the NDV LaSota strain. * *p* < 0.05, ** *p* < 0.01.

Regarding TNF-α, the results were similar to the results of transcriptome sequencing. No significant difference was detected in the infection groups compared with the control groups from 2 h to 8 h (*p* > 0.05). However, from 14 h to 24 h post-infection, the expression levels of TNF-α in the infection groups were obviously higher than those in the control groups (*p* < 0.01) (Figure 5A). Regarding IL-17, consistent with the results of transcriptome sequencing, its expression levels in the infection groups were significantly higher than those in the control groups from 2 h to 24 h (*p* < 0.05) (Figure 5B). Regarding IL-1β, its expression levels distinctly increased from 4 h to 6 h and 14 h to 16 h after BHK-21 cells were infected with the NDV LaSota strain (*p* < 0.01), but they significantly declined at 8 h, 10 h, 20 h, and 24 h (*p* < 0.05) (Figure 5C). Regarding IL-6, its expression levels were consistent with the results of transcriptome sequencing. From 2 h to 8 h and 16 h to 24 h after BHK-21 cells were infected with the NDV LaSota strain, the expression levels of IL-6 were significantly higher compared with the control groups (*p* < 0.05), but they obviously decreased from 10 h to 14 h post-infection (*p* < 0.05) (Figure 5D). Regarding IL-10, only at 16 h after BHK-21 cells were infected with the NDV LaSota strain, its expression level was obviously higher than that in the control group (*p* < 0.01). However, at other times, the expression levels of IL-10 in the infection groups were significantly lower than those in the control groups (*p* < 0.05) (Figure 5E).

### 3.6. Inhibition of TLR3-TRIF-NF-κB Signaling Pathway Promoted Proliferation of NDV LaSota Strain in BHK-21 Cells

While BHK-21 cells were infected with the NDV LaSota strain, the TLR3 inhibitor, TRAF3 inhibitor, and NF-κB inhibitor were respectively used. Regarding the logHA values and lgTCID_50_ values, there was no significant difference between the BHK + TLR3 inhibitor group and the BHK + NFκB inhibitor group (*p* > 0.05). Moreover, there was no significant difference between the BHK + TRAF3 inhibitor group and the BHK-21 cells control group (*p* > 0.05). But the logHA values and lgTCID_50_ values in the BHK + TLR3 inhibitor group and the BHK + NFκB inhibitor group were significantly higher than those in the BHK + TRAF3 inhibitor group and the BHK-21 cells control group (*p* < 0.05) (Table 3). The results suggested that inhibition of either the TLR3-signaling pathway or the NF-κB-signaling pathway can obviously promote proliferation of the NDV LaSota strain in BHK-21 cells, thereby increasing the viral titers.

### 3.7. Successful Construction of BHK-TLR3^−^ Cells with TLR3 Knocked out by CRISPR/Cas9 System

**Figure 6:**
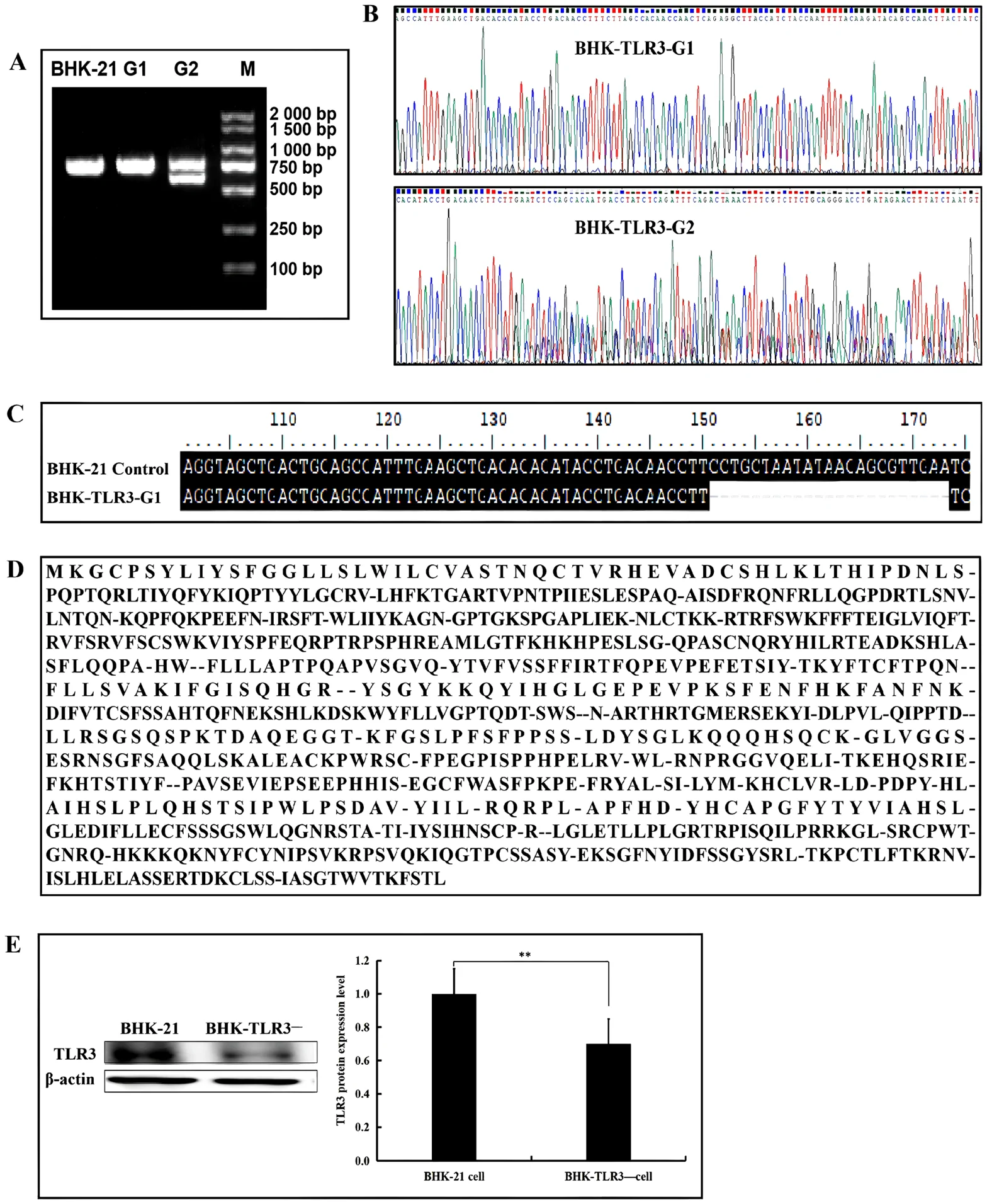
Identification of BHK-TLR3^−^ cells. (**A**) RT-PCR analysis of TLR3 in the BHK-TLR3-G1cell clone and the BHK-TLR3-G2 cell clone. (**B**) Sequencing peak chart of TLR3 in the BHK-TLR3-G1 cell clone and the BHK-TLR3-G2 cell clone. (**C**) Sequence alignment of the mutant TLR3 in the BHK-TLR3-G1 cell clone with the wild-type TLR3 sequence. “一” represents deletion in the BHK-TLR3-G1 cell clone. (**D**) Predicted amino acid sequence of the mutant TLR3 gene in the BHK-TLR3-G1 cell clone. (**E**) The protein expression level of TLR3 in the BHK-TLR3^−^ cells and analysis of grayscale value by Western blot. ** *p* < 0.01.

To verify successful CRISPR/Cas9-mediated disruption of the TLR3 gene, two BHK-TLR3^−^ cell clones (BHK-TLR3-G1 and BHK-TLR3-G2) were analyzed by RT-PCR and Sanger sequencing. The results of RT-PCR showed that the target gene was successfully obtained in the BHK-TLR3-G1 cell clone, the BHK-TLR3-G2 cell clone, and BHK-21 cells (gene length: 758 bp), but the BHK-TLR3-G2 cell clone exhibited two bands (Figure 6A). Furthermore, the sequencing peak graph of the BHK-TLR3-G2 cell clone displayed a double peak, indicating that the cell clone is heterozygous (Figure 6B). However, compared with the wild-type BHK-21 cells, the BHK-TLR3-G1 cell clone displayed a single peak and missed a 23 bp gene fragment at the sgRNA site (Figure 6C). Therefore, the BHK-TLR3-G1 cell clone was named BHK-TLR3^−^ cells for further study. Subsequently, the predicted TLR3 protein sequence showed that the mutant TLR3 encodes 898 amino acids (TLR3 full-length: 905 amino acids), and some codons cannot be translated normally (Figure 6D). It may be that the deletion of a 23 bp gene fragment leads to frameshift mutations in the reading frame, thereby resulting in the non-translation of subsequent codons. Finally, the expression level of TLR3 protein in BHK-TLR3^−^ cells was detected via Western blot analysis. The results indicated that the expression level of TLR3 protein in BHK-TLR3^−^ cells was significantly lower than that in BHK-21 cells (*p* < 0.01) (Figure 6E). These findings confirm the successful generation of TLR3-mutant BHK-TLR3^−^ cells.

### 3.8. Morphology Observation of BHK-TLR3^−^ Cells

**Figure 7:**
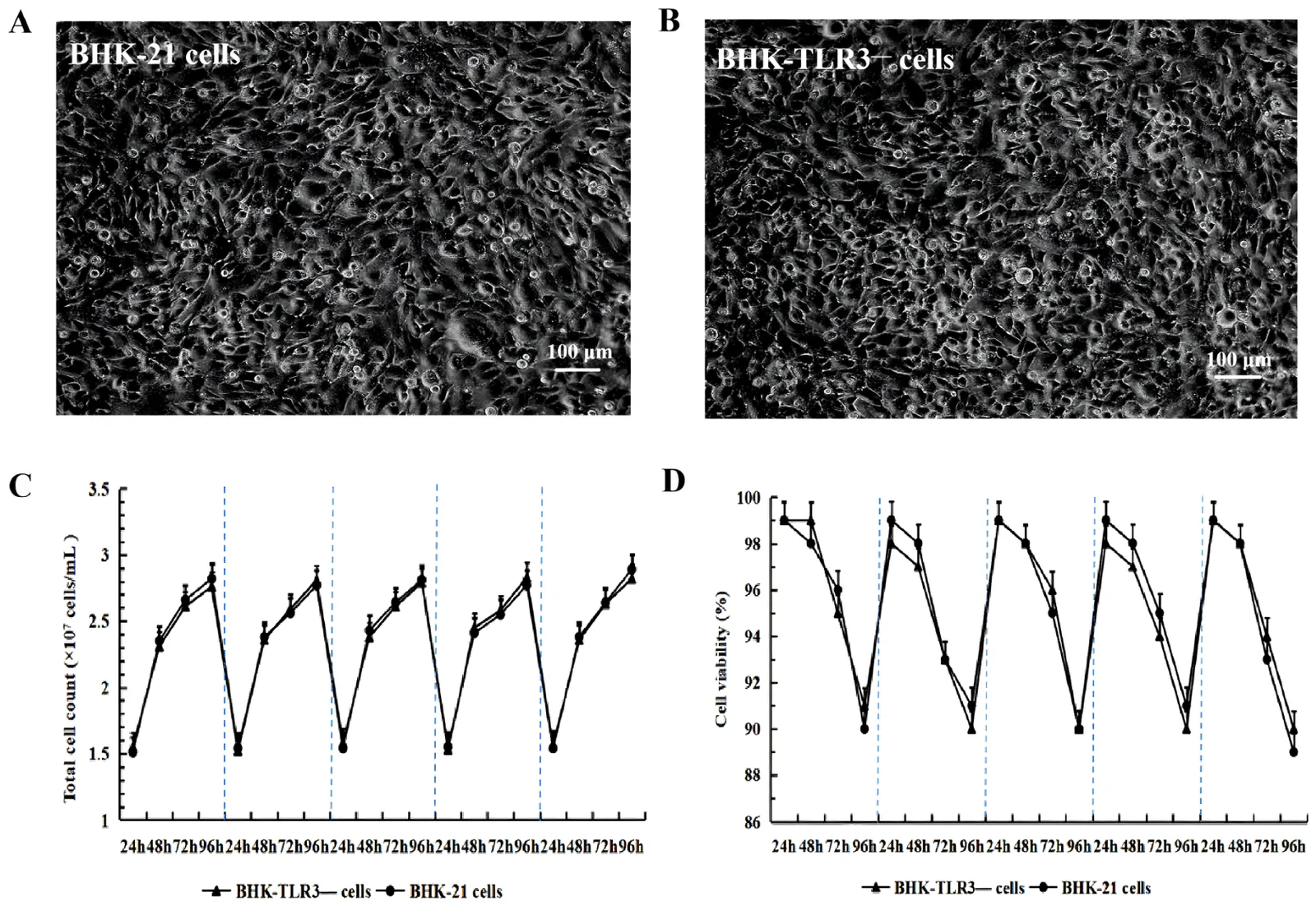
Growth characteristics of BHK-TLR3^−^ cells. (**A**) Morphology of wild-type BHK-21 cells (original magnification, ×200). (**B**) Morphology of BHK-TLR3^−^ cells (original magnification, ×200). (**C**) Growth kinetics of BHK-TLR3^−^ cells and BHK-21 cells. (**D**) Cell viability of BHK-TLR3^−^ cells and BHK-21 cells.

Following TLR3 gene mutation, the BHK-TLR3^−^ cells displayed a typical fusiform shape at 48 h post-passage (Figure 7A,B), similar to the wild-type BHK-21 cells. The result indicated that loss of TLR3 does not affect morphology of the BHK-TLR3^−^ cells.

### 3.9. Growth Kinetics and Cell Viability of BHK-TLR3^−^ Cells

During culture of BHK-TLR3^−^ cells and BHK-21 cells, the cell density and viability were determined. The results displayed that BHK-TLR3^−^ cells and BHK-21 cells did not exhibit comparable growth kinetics and viability at 48 h after passage (*p* > 0.05) (Figure 7C,D). At 96 h after passage, though the cell densities of both cells slightly rose, their viability declined to approximately 90% (*p* > 0.05) (Figure 7D). These results indicated that the growth kinetics and cell viability of the BHK-TLR3^−^ cells do not change compared with the BHK-21 cells.

### 3.10. Stability of TLR3 Mutant Gene in BHK-TLR3^−^ Cells

After BHK-TLR3^−^ cells were continuously cultured and passaged up to the 20th generation, the sequence similarity of the mutant TLR3 gene was analyzed. The results showed that the TLR3 mutant gene of BHK-TLR3^−^ cells remained 100% similar between the 0th, 10th, and 20th generations, without evidence of revertant mutations.

### 3.11. BHK-TLR3^−^ Cells Promote Proliferation of NDV LaSota Strain

The HA titer and TCID_50_ value were measured approximately 16 h after BHK-21 cells and BHK-TLR3^−^ cells were infected with the NDV LaSota strain. The results displayed that the TCID_50_ and HA values in the BHK-TLR3^−^ cells group were significantly higher than those in the BHK-21 cells group (*p* < 0.01), indicating that the BHK-TLR3^−^ cells can effectively promote the proliferation of the NDV LaSota strain, thereby increasing the viral titer (Table 4).

### 3.12. ELISA Detection of IFN-α, IFN-β, IL-6, IL-1β, and TNF-α After the BHK-TLR3^−^ Cells Were Infected with the NDV Lasota Strain

**Figure 8:**
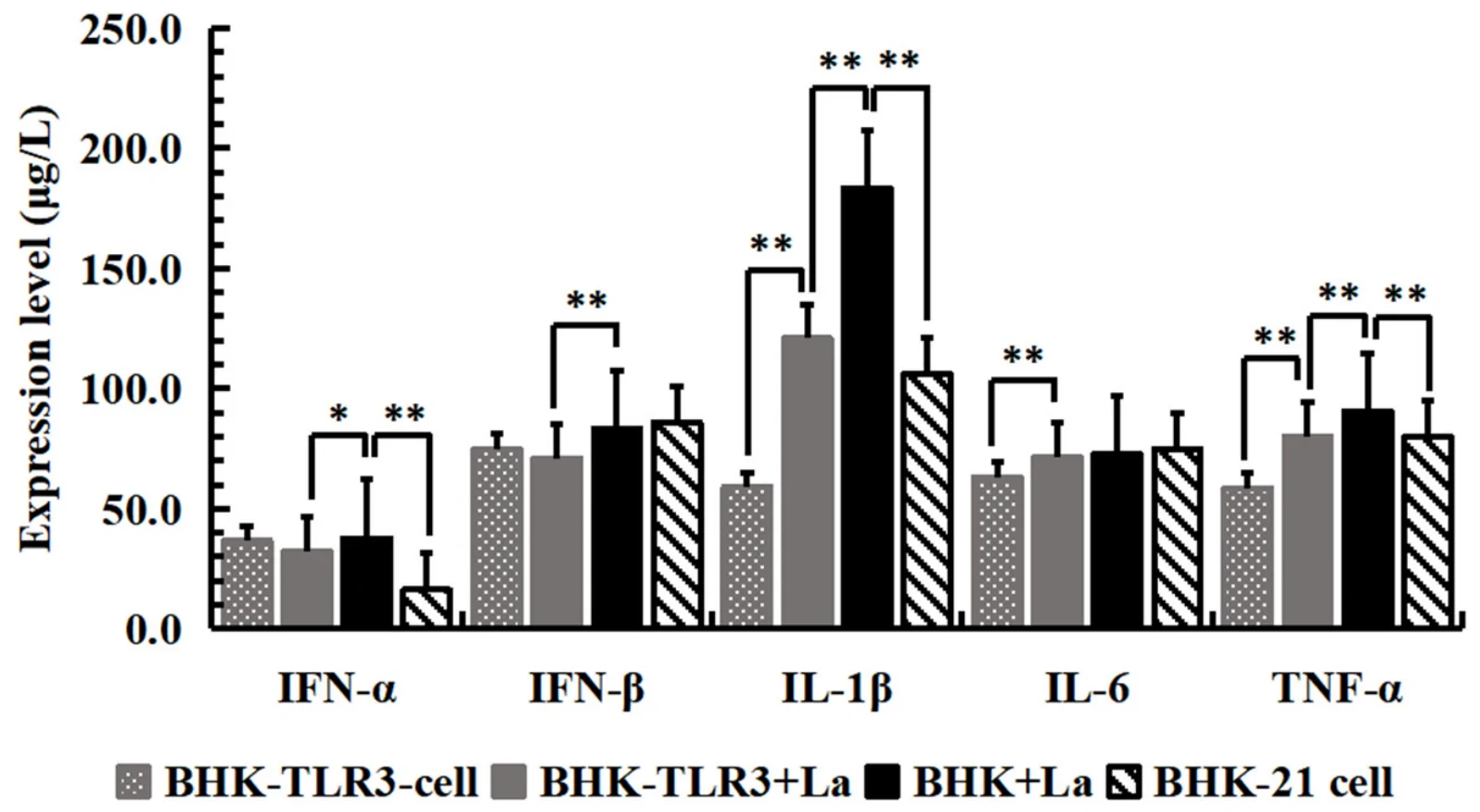
The ELISA detection for IFN-α, IFN-β, IL-1β, IL-6, and TNF-α after BHK-TLR3^−^ cells were infected with the NDV LaSota strain. * *p* < 0.05, ** *p* < 0.01.

Regarding type I interferon, the expression levels of IFN-α and IFN-β in BHK-TLR3 + La groups were not significantly different compared with the BHK-TLR3^−^ cells control groups (*p* > 0.05), but they all obviously decreased compared to those in the BHK + La groups (*p* < 0.01). Regarding pro-inflammatory cytokines IL-1β and IL-6, their expression levels in the BHK-TLR3 + La groups were obviously higher than those in the BHK-TLR3^−^ cells control groups (*p* < 0.01). But compared with the BHK + La groups, the expression level of IL-1β in the BHK-TLR3 + La group was significantly decreased (*p* < 0.01), and the expression level of IL-6 in the BHK-TLR3 + La group was not significantly different (*p* > 0.05). Regarding TNF-α, its expression levels in the BHK-TLR3 + La group were significantly higher than those in the BHK-TLR3^−^ cells control groups (*p* < 0.01), but were obviously decreased compared to those in the BHK + La groups (*p* < 0.01). These results showed that knockout of the TLR3 gene significantly inhibited the expression of IFN-α, IFN-β, IL-1β, and TNF-α after BHK-TLR3^−^ cells were infected with the NDV LaSota strain, especially the IL-1β and TNF-α (Figure 8).

## 4. Discussion

NDV is a highly infectious virus that can lead to huge economic losses to the poultry industry [25,26]. NDV can infect a wide range of cell types, but the relatively low viral titer remains a significant barrier to large-scale production with cell lines. This may be due to the species-specific selectivity of host cells by different NDV strains [20,27], or it might be due to the varying antiviral immune responses produced by different host cells. How can the proliferation of NDV within cells be promoted, thereby increasing virus titer? It has been reported that knocking out the MDA5 and TLR3 receptors of DF-1 cells can significantly weaken the natural immune response of host cells and promote the proliferation of influenza virus in cells [28]. What is the main innate immune response induced by NDV in BHK-21 cells? Can the knockout of relevant innate immune genes promote the proliferation of NDV? These questions are worth exploring.

The PRRs of poultry that recognize RNA viruses mainly include TLR3, TLR7, TLR8, and MDA5, etc. [29]. Among them, TLR3 primarily recognizes the nucleic acids of pathogens through the MyD88-independent signaling pathway (TRIF signaling pathway) [11]. After the TLR3 receptor is activated, TRIF transiently colocalizes with TLR3. Then, it dissociates from the receptor and forms a speckle-like structure that relocalizes with downstream-signaling molecules such as tumor necrosis factor receptor-associated factor 2 (TRAF2), TRAF6, and receptor-interacting protein-1 (RIP-1). Subsequently, with the cooperation of TRAF3, NF-κB activator-binding kinase 1 (TBK1), inhibitor of κB (IκB) kinase-related kinase-ε, and NF-κB-activating kinase-associated protein 1 (NAP1), this signaling pathway ultimately activates transcription factors—namely, IFN-regulatory factor 3/7 (IRF3/7), the TRIF-dependent NF-κB, and the activator protein 1 (AP-1)—thus mediating the production of type I IFNs, pro-inflammatory cytokines, and chemokines, respectively. It has been reported that the NDV LaSota strain promotes the expression of TLR3, TLR7, and MDA5 genes in DF-1 cells, but the virulent NDV inhibits the expression of TLR2, TLR3, TLR4, and TLR7 in chicken embryo fibroblasts [30,31]. In this study, we demonstrated that the NDV LaSota strain obviously promoted the expression of TLR3, TRIF, and NF-κB in BHK-21 cells from 2 h to 24 h after infection, but the expression levels of TRAF3 in the infection groups were obviously higher than those in the control group only from 14 h to 24 h. Combined with the detection of cytokine expression levels, we concluded that NDV mainly induced the TLR3-TRIF-NF-κB signaling pathway in BHK-21 cells, and the TLR3-TRIF-TRAF3 signaling pathway only revealed its effect in the later stage of infection. On the other hand, although the expression levels of NOD2 were significantly higher after BHK-21 cells were infected with the NDV LaSota strain, they were distinctly lower than the expression levels of TLR3. Additionally, the gene expression levels of TLR7 and TLR8 displayed fluctuating changes. All these results indicate that the signaling pathways of innate immunity induced by NDV in different cells are distinct, which may be related to the NDV virulence and cell types.

In order to explore whether inhibiting the signaling pathway induced by TLR3 can significantly promote the proliferation of NDV in BHK-21 cells, the TLR3 inhibitors, TRAF3 inhibitors, and NF-κB inhibitors were respectively employed while BHK-21 cells were infected with the NDV LaSota strain. The results show that inhibition of the signaling pathway regulated by TLR3 can significantly promote the proliferation and increase the viral titer of the NDV LaSota strain in BHK-21 cells. On the other hand, the results once again demonstrate that the TLR3-TRIF-NF-κB signaling pathway induced by the NDV LaSota strain is the main innate immune response in BHK-21 cells. Above all, these results lay a foundation for our subsequent construction of BHK-21 cell lines deficient in innate immune-related genes. Therefore, we successfully constructed a TLR3 gene-knockout BHK-21 cell clone (BHK-TLR3^−^ cells) using the CRISPR/Cas9 system. Compared with BHK-21 cells, the morphology and growth characteristics of BHK-TLR3^−^ cells remain unchanged. But the BHK-TLR3^−^ cells can significantly promote the proliferation of NDV LaSota strain, resulting in an increased viral titer compared with BHK-21 cells. This might be due to the fact that the knockout of the TLR3 gene suppresses the antiviral immune response of BHK-21 cells to some extent. However, in the present study, all the experiments were performed in adherent culture. In suspension culture, it remains to be investigated whether the BHK-TLR3^−^ cells can still possess the ability to promote proliferation of the NDV LaSota strain. Therefore, future work will be focused on achieving the full suspension culture of BHK-TLR3^−^ cells and further determining its potential abilities to enhance the viral titer of the NDV LaSota strain.

## 5. Conclusions

Our study demonstrates that the TLR3-TRIF-NF-κB signaling pathway is the main innate immune response after BHK-21 cells were infected by the NDV LaSota strain. However, inhibition of the signaling pathway can obviously promote the proliferation of NDV in BHK-21 cells. Therefore, we successfully constructed the BHK-TLR3^−^ cells with the TLR3 gene knocked out. Notably, the NDV LaSota strain propagated in BHK-TLR3^−^ cells achieved significantly higher viral titers compared with BHK-21 cells. These results provide a promising foundation for the development of NDV production using cell culture platforms.

## Figures and Tables

**Table 1:** The primers used in the qRT-PCR reaction.

Gene	Forward Sequence (5′-3′)	Reverse Sequence (5′-3′)	GenBank Accession No.
β-actin	CTGGACTTCGAGCAAGAGATG	CCACAGGATTCCATACCCAGG	101822713
TLR3	GAGCAAGAACTCACCGGACA	ACCCTCCTGAGCATCAGTCT	101843286
TLR7	GTCCTTGGGTCTCGATGGTT	CAGTCCACGATCACATGGGT	101840275
TLR8	TGAGCCACAACGCACACTAT	TCAAGGCGGTTTCCACTGAA	101840550
NOD2	AAGGGAACGGGGATTCCTGA	TTCACTGTGGCGAGGTCAAG	101838071
TRAF3	GCCGTTGAGAGGTCCTACAC	GTGGCCATAAGCAGTCACCT	101831388
TRIF	CTGGGGGTCAACAACCACAT	ACTTTAGCCAAGTGGGCTCC	101834348
NF-κB	TACACCTGCAGTTTGACGCT	CTGTGGGGTGAGACATGGAC	101831177

**Table 2:** The sgRNA and identification primers of the TLR3 gene.

Name	Forward/Reverse Sequence (5′–3′)
sgRNA	CCTCATGTCTCACCGTACATTGG
sgRNA reverse transcription primer	TAATACGACTCACTATAGGGCCTCATGTCTCACCGTACATTGGGTTCAGAGCTATGCTGGA
TLR3 PCR identification primer	TLR3-Forward: TGCTTGGTGCGTTACTCTATGG TLR3-Reverse: CACATCCCAGCCATCAGGAAA

**Table 3:** The viral titers of NDV LaSota strain in BHK-21 cells processed by the TLR3-signaling pathway inhibitors.

Cell Type	Viral Titer	
	logHA	lgTCID_50_/0.1 mL
BHK + TLR3 inhibitor	10.30 ± 0.60 *	7.38 ± 0.13 **
BHK + TRAF3 inhibitor	7.30 ± 0.60	6.00 ± 0.10
BHK + NFκB inhibitor	10.70 ± 1.20 *	7.13 ± 0.13 **
BHK-21 cells	8.00 ± 1.00	6.13 ± 0.13

* *p* < 0.05. ** *p* < 0.01.

**Table 4:** Viral titer in BHK-TLR3^−^ cells after NDV LaSota strain infection.

Cell Type	Viral Titer	
	logHA	lgTCID_50_/0.1 mL
BHK-TLR3^−^ cells	11.00 ± 0.60 **	7.67 ± 0.19 **
BHK-21 cells	8.00 ± 0.00	6.29 ± 0.14

** *p* < 0.01.

## Data Availability

Data will be made available on request.

## Abbreviations

The following abbreviations are used in this manuscript:

ND	Newcastle disease
NDV	Newcastle disease virus
DMEM	Dulbecco’s Modified Eagle Medium
PRRs	Pattern recognition receptors
TLRs	Toll-like receptors
TCID_50_	Tissue culture infectious dose
sgRNA	Single-guide RNA
TRAF2	Tumor necrosis factor receptor-associated factor 2
RIP-1	Receptor-interacting protein-1
TBK1	NF-κB activator-binding kinase 1
IκB	Inhibitor of κB
NAP1	NF-κB-activating kinase-associated protein 1
IRF3/7	IFN-regulatory factor 3/7
AP-1	Activator protein 1
